# Infant Mortality Rates for Farming and Unemployed Households in the Japanese Prefectures: An Ecological Time Trend Analysis, 1999–2017

**DOI:** 10.2188/jea.JE20190090

**Published:** 2021-01-05

**Authors:** Mariko Kanamori, Naoki Kondo, Yasuhide Nakamura

**Affiliations:** 1 Department of Health and Social Behavior and Department of Health Education and Health Sociology, The University of Tokyo, Tokyo, Japan; 2 School of Nursing and Rehabilitation, Konan Women’s University, Hyogo, Japan

**Keywords:** infant mortality, health inequality, occupation, farmer, unemployed worker

## Abstract

**Background:**

Recent research suggests that Japanese inter-prefecture inequality in the risk of death before reaching 5 years old has increased since the 2000s. Despite this, there have been no studies examining recent trends in inequality in the infant mortality rate (IMR) with associated socioeconomic characteristics. This study specifically focused on household occupation, environment, and support systems for perinatal parents.

**Methods:**

Using national vital statistics by household occupation aggregated in 47 prefectures from 1999 through 2017, we conducted multilevel negative binomial regression analysis to evaluate occupation/IMR associations and joinpoint analysis to observe temporal trends. We also created thematic maps to depict the geographical distribution of the IMR.

**Results:**

Compared to the most privileged occupations (ie, type II regular workers; including employees in companies with over 100 employees), IMR ratios were 1.26 for type I regular workers (including employees in companies with less than 100 employees), 1.41 for the self-employed, 1.96 for those engaged in farming, and 6.48 for unemployed workers. The IMR ratio among farming households was 1.75 in the prefectures with the highest population density (vs the lowest) and 1.41 in prefectures with the highest number of farming households per 100 households (vs the lowest). Joinpoint regression showed a yearly monotonic increase in the differences and ratios of IMRs among farming households compared to type II regular worker households. For unemployed workers, differences in IMRs increased sharply from 2009 while ratios increased from 2012.

**Conclusions:**

Inter-occupational IMR inequality increased from 1999 through 2017 in Japan. Further studies using individual-level data are warranted to better understand the mechanisms that contributed to this increase.

## INTRODUCTION

The infant mortality rate (IMR), the number of deaths of children under 1 year of age per 1,000 live births in the same year, is an important measure of population health and serves as an indicator of the effect of economic and social conditions on the health of mothers and newborns.^1^^,^^2^ Japan, like many other wealthy, developed countries, has a low IMR.^3^ Similarly, the mortality risk between 0 and 5 years old was the lowest level in the world at the time.^4^ However, these risks are not necessarily homogeneous across the regions of Japan. For example, Nagata et al (2017) recently reported that inter-prefecture inequality in child mortality had increased since the 2000s.^5^ This increase in inequality in regional child mortality may be linked to changes in some of the social determinants of child mortality observed across high-income countries that include relative poverty, income inequality and social policies, such as workplace maternal leave policies.^6^^–^^8^ As the relative poverty rate for children in Japan increased by 1.5 times from 1985 to 2012, it is possible that an expansion in social and economic differences might also be affecting the IMR and increasing regional inequalities.^6^^,^^9^

The health of pregnant women and infants is also greatly affected by their socioeconomic status, including household income and occupation. Sidebotham et al have suggested for instance, that factors affecting child and adolescent mortality in high-income countries “can be conceptualized within four domains—intrinsic (biological and psychological) factors, the physical environment, the social environment, and service delivery. The most prominent factors are socioeconomic gradients…”.^7^ If a pregnant woman is working, job stress and the number of hours worked, as well as the work environment, available medical services, and workplace support might all affect her health and that of her unborn child. For example, in Japan paid maternity leave and childcare leave benefits depend on employment conditions; moreover, there is no paid maternity leave for self-employed people and farmers.^10^ In relation to this, in an earlier study Nishi and Miyake (2007) calculated IMR ratios across occupations at the national level and found that the IMR in unemployed households was 4.2 times higher than the rate in all employed Japanese households from 1995 to 2004.^11^ However, since Nishi and Miyake’s study, to the best of our knowledge, there has been no research on the occupation-IMR association that has covered more recent years or focused on smaller geographical units, such as prefectures. In addition, there have been no studies that have examined how the regional characteristics of each prefecture and social factors of each household interact to affect the IMR. This regional influence is potentially very important, as the environment of pregnant women and infants may differ depending on regional characteristics, even when such women are working in the same occupation.

Therefore, in this ecological study, which uses time-trend data for Japanese prefectures from 1999 through 2017, we aimed to clarify the trends in inter-occupational inequality in infant mortality and generate hypotheses about the relationship between macro-level social status and the IMR. To clarify the association between regional characteristics and inequality in the IMR, we also examined the interaction effects between rurality and industry structure, such as the prevalence of farming and of different household occupations within each prefecture. We hypothesized that the association between infant mortality and household occupation would vary in relation to a prefecture’s level of rurality and regional industry structure, as this was likely to be reflected in differing social circumstances, such as the availability of accessible medical resources that could potentially affect the link between infant mortality and household occupation.

## METHODS

### Data

We obtained prefectural data on infant births and deaths aggregated by household occupation.^12^ These vital statistics data are publicly available for the entire Japanese population between 1999 and 2017. When an infant is born or dies, a parent or household member must notify the infant’s municipality of residence or the place where the infant was born or died. We also used government prefectural summary statistics from 2000, 2005, 2010, and 2015.^13^ As the prefectural data are only collected and published once every 5 years, we linked the prefectural data from 2000, 2005, 2010, and 2015 to infant mortality data from 1999 to 2002, 2003 to 2007, 2008 to 2012, and 2013 to 2017, respectively. To visually depict the nationwide regional distribution of infant mortality, we also used governmental geospatial information and nationwide municipal boundary data from the Environmental Systems Research Institute, Japan.^14^

### Measurements

#### Main outcome (IMR)

The main outcome was the prefectural IMR by household occupation.

#### Explanatory variables (household occupation)

Japanese vital statistics for infant births and deaths include information about household occupation that is provided by a family member in response to a query about “the main household occupation.” The family member chooses one of six household occupation categories: (i) type I regular worker, (ii) type II regular worker, (iii) self-employed, (iv) farming, (v) other, and (vi) unemployed. “Type I regular worker” includes employees in small companies with fewer than 100 employees. “Type II regular worker” refers to those working in medium to large companies with more than 100 employees, executives, and government officials. The “Self-employed” comprise those who are engaged in running their own companies/businesses (ie, working freelance). The “Farming” category refers to workers either engaged solely in agriculture or both in agriculture and other professions. The “Other” category consists of workers employed for a continuous period of less than one year. The “Unemployed” category includes households in which nobody is employed. Missing data were included in the “unknown” category.

#### Other variables

We used population density as a proxy measure of rurality in each prefecture. As the IMR in farming households was higher than for other occupations when we calculated the descriptive statistics, we also evaluated the relative predominance of farming in each prefecture, measuring farm density (number of farming households per 100 households) and used it as a proxy measure of the industrial structure in each prefecture. In order to make the regression analysis estimates comparable, we standardized these two measures by their overall means and standard deviation.

### Statistical analysis

#### Thematic maps

We created a visual representation of the nationwide regional IMR distribution by household occupation using thematic maps of prefectural data. We calculated the IMR by dividing total infant mortality for the years 1999 through 2017 by the total number of births for the same period. We used Arc GIS 10.5 (Esri, Redlands, CA, USA) to create the thematic maps.^15^

#### Calculation of IMR by household occupation

We calculated IMR ratios by household occupation using multilevel negative binomial regression analysis that took into account the hierarchical structure of the time series data. Goodness-of-fit statistics and residual plots of the Poisson or negative binomial regression models confirmed that the IMR followed a negative binomial distribution. The level 1 variable was the yearly IMR by household occupation (*N* = 7 * 19 * 47 = 6,251) between 1999 and 2017, while the level 2 variable was the prefecture (*N* = 47). We used the *menbreg* command in STATA/MP 15.1 (Stata Corp, College Station, TX, USA) and incorporated a random intercept at the prefecture level in each model, specifying robust variance.^16^ To estimate the IMR, we set the number of deaths of children under 1 year of age as the outcome variable and the number of living births for each unit as the offset variable. We also adjusted for dummy year variables (model 1). Prefectural population density and farm density in each year were added as covariates (model 2). Further, to assess how the association between the main household occupation and IMRs varied by prefectural characteristics, we also included interaction terms between population density and occupation (model 3) and between farm density and occupation (model 4).

#### Health inequality measures and trend analysis

Next, we calculated health inequality measures for the data with unordered social groups, such as the rate difference and rate ratio compared to the most privileged occupation (type II regular worker), between group variance, the index of disparity, mean log deviation, and the Theil index, using the Health Disparity Calculator version 1.2.4.^17^ The rate difference and between group variance represent the absolute amount of inequality, while the rate ratio, index of disparity, mean log deviation, and Theil index represent the level of inequality in relative terms. The *index of disparity* represents changes in health inequality regardless of which group changes, whereas the *mean log deviation* is sensitive to changes among the least healthy group. Theil index characteristics are similar to the mean log deviation, but Harper and Lynch have suggested that the Theil index is slightly more influenced by high mortality ratios whereas the mean log deviation is slightly more influenced by large population shares.^18^ Detailed information about these measures is available elsewhere.^18^^,^^19^ Then, we plotted these inequality measures and visually evaluated the secular trends. To evaluate the changes in these inequality measures, we conducted joinpoint analysis using the Joinpoint Regression Program version 4.6.0.0 (National Cancer Institute, Bethesda, MD, USA), to explore the potential mathematical points of change in trends.^20^ We tested whether or not an apparent change in a trend was statistically significant using grid search methods that created a “grid” of all possible locations for joinpoints (ie, the points connecting two different trend slopes) and calculated the sum of the squared error at each point to find the best possible fit. The program performs permutation tests to select the number of joinpoints. Using each health inequality measure as a dependent variable and the year as an independent variable, we estimated joinpoints and their confidence intervals. Further details of joinpoint regression analysis are available elsewhere.^21^^,^^22^ Our preliminary analysis identified two occupational categories that had especially high IMRs for this trend analysis—farmers and the unemployed—so we focused on these two groups.

### Ethical approval

As this study used only publicly available secondary aggregated data, a formal ethics review was not required, as per the guidelines of the Graduate School of Medicine and Faculty of Medicine at the University of Tokyo.^23^ We obtained a formal confirmation of this from the Ethics Review Board of the Graduate School of Medicine and Faculty of Medicine at the University of Tokyo (2018117NIe).

## RESULTS

**Figure 1.**
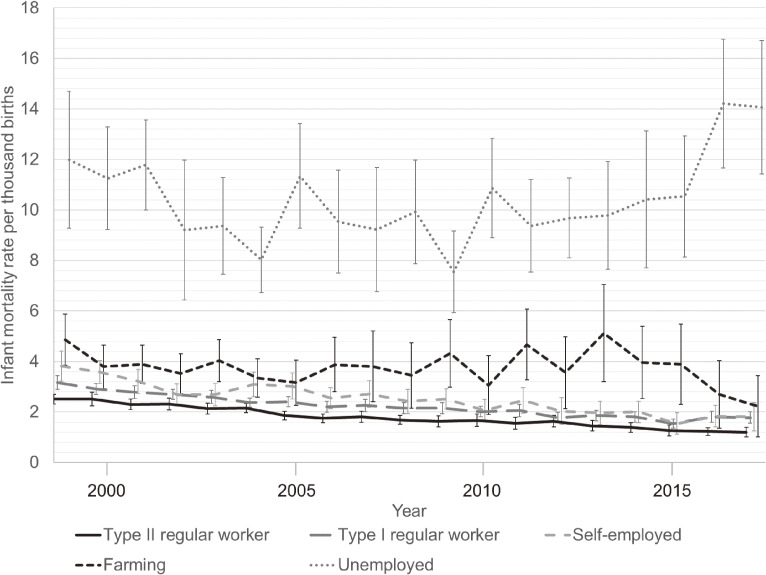
Trends in the infant mortality rate by occupation in each household in Japan, 1999–2017. Error bars indicates 95% confidence intervals.

**Figure 2.**
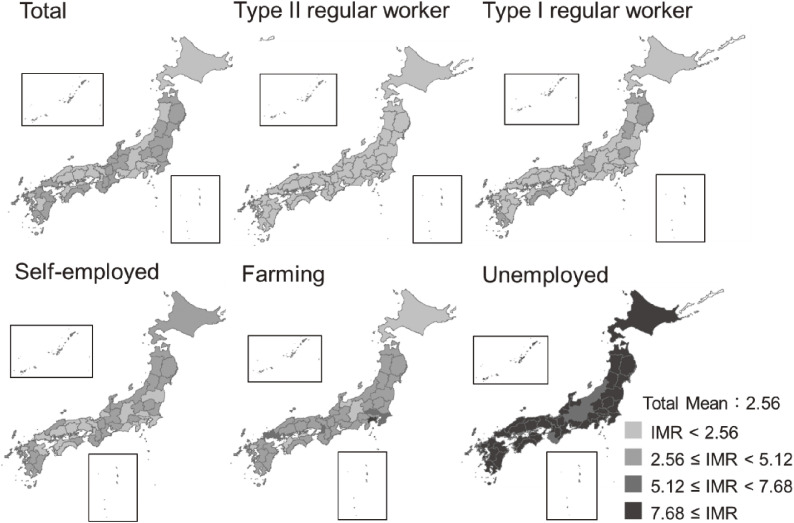
Geographical distribution of mean infant mortality rates (IMR) per 1,000 births by household occupations in Japanese prefectures, 1999–2017

Descriptive statistics showed that the number of births was highest and the IMR lowest among type II regular workers, and that the IMR continued to decrease across the 19-year period (Table 1, eTable 1 and Figure 1). In contrast, IMRs of the farming group increased in relative terms. The unemployed had the highest IMRs among the occupational categories, which reached their highest point during the 2016 observation period. In almost all prefectures, the IMRs of type II regular workers were below the national average (Figure 2). In prefectures with large populations, such as Kanagawa Prefecture and Osaka Prefecture, the IMR rate among farmers was more than twice the total mean rate.

**Table 1.** The number of infant deaths and births by occupation in each household in Japan, in 2000, 2005, 2010, and 2015 (full data are available in the supplemental figure)

Occupation	2000		2005		2010		2015	
	Death	Birth	Death	Birth	Death	Birth	Death	Birth
Type II regular worker	1,126	484,043	780	421,978	700	448,336	561	458,419
Type I regular worker	1,140	411,579	911	380,029	676	373,397	535	334,744
Self-employed	344	100,831	241	84,615	158	78,939	103	71,771
Farming	129	36,294	77	23,872	53	18,459	44	13,147
Other	530	117,250	417	99,146	354	96,440	267	84,501
Unemployed	360	23,452	286	23,742	259	24,081	211	18,535
Unknown	193	16,888	239	28,978	247	31,527	194	24,507

Total	3,822	1,190,337	2,951	1,062,360	2,447	1,071,179	1,915	1,005,624

Regression analysis showed that in comparison to type II regular workers, the IMR ratio was 1.26 for type I regular workers, 1.41 for the self-employed, 1.96 for those engaged in farming, and 6.48 for the unemployed (Table 2). Between-prefecture variance decreased from 0.024 in the null model to 0.007 in the adjusted model (model 1). These results remained largely unchanged after adjusting for population density and farm density (model 2).

**Table 2.** Ratios of infant mortality rates with 95% confidence intervals: results of multilevel negative binomial regression analysis

	Model 1	Model 2	Model 3	Model 4
**Occupations in each household**				
Type II regular worker	Reference			
Type I regular worker	1.26 [1.22, 1.29]	1.26 [1.22, 1.29]	1.25 [1.21, 1.29]	1.24 [1.20, 1.28]
Self-employed	1.41 [1.35, 1.46]	1.41 [1.35, 1.46]	1.43 [1.38, 1.49]	1.41 [1.35, 1.47]
Farming	1.96 [1.85, 2.08]	1.96 [1.75, 2.20]	1.98 [1.78, 2.21]	1.95 [1.72, 2.21]
Other	2.30 [2.22, 2.38]	2.30 [2.12, 2.50]	2.18 [2.05, 2.32]	2.17 [2.04, 2.32]
Unemployed	6.48 [6.24, 6.74]	6.48 [5.70, 7.38]	5.75 [5.27, 6.29]	5.49 [5.08, 5.93]
**Population density**^a^		0.99 [0.93, 1.05]	0.94 [0.89, 1.00]	0.99 [0.92, 1.05]
**Farm density**^a^		0.99 [0.94, 1.05]	0.99 [0.94, 1.04]	1.05 [0.99, 1.11]
**Population density × occupations**				
Type II regular worker			Reference	
Type I regular worker			1.00 [0.98, 1.02]	
Self-employed			0.98 [0.96, 1.00]	
Farming			1.22 [1.07, 1.40]	
Other			1.10 [1.06, 1.15]	
Unemployed			1.19 [1.14, 1.25]	
**Farm density × occupations**				
Type II regular worker				Reference
Type I regular worker				0.98 [0.96, 1.01]
Self-employed				1.00 [0.97, 1.04]
Farming				0.87 [0.77, 0.98]
Other				0.88 [0.83, 0.94]
Unemployed				0.77 [0.71, 0.82]

**Random parameters:** Between-prefecture variance^b^ Null:0.024 [0.006]				
	0.007 [0.002]	0.007 [0.002]	0.007 [0.002]	0.008 [0.002]

^a^Standardized value.

^b^Standard error in parentheses.

Covariates: Year (dummy variables), Offset variable: number of births.

The category of “Unknown” was taken into account in all variables including interaction terms.

**Figure 3.**
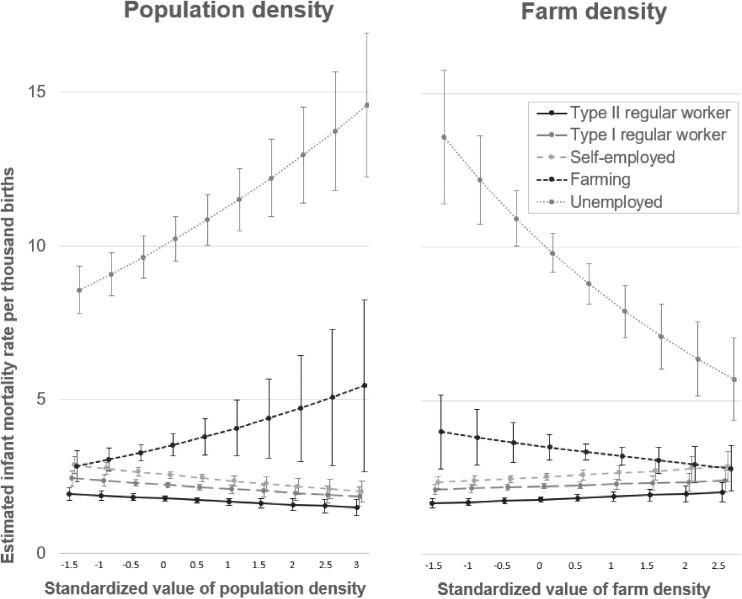
Estimated infant mortality rate per thousand births from the models with interactions terms (models 3 and 4 in Table 2). Error bars indicate 95% confidence intervals.

The interaction terms between population density and three occupation categories—farming, other, and unemployed—were statistically significant, showing high IMRs in those households in prefectures with a high population density. In contrast, these three occupation categories also had smaller IMRs in the prefectures with high farm densities (models 3 and 4 in Table 2). IMRs estimated from the models with interaction terms indicated that the IMRs for the farming and unemployed categories were much higher than for the other categories and increased as population density increased but decreased as farm density increased (Figure 3).

**Figure 4.**
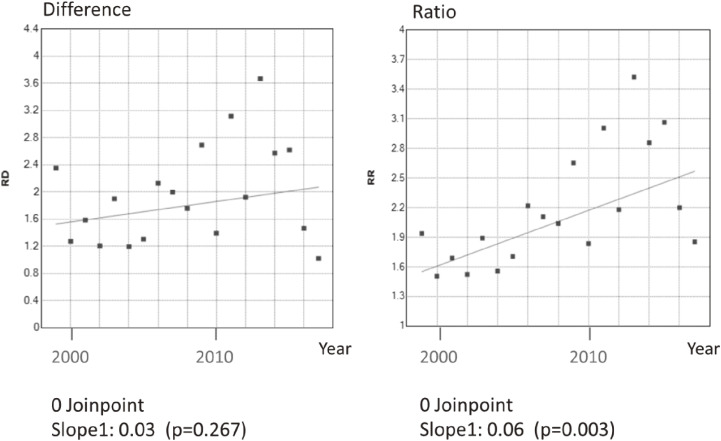
Trends in the Rate Differences (RD) and the Rate Ratios (RR) of infant mortality between farming and type II regular worker household in Japan, 1999–2017. The dots mean each value and the lines mean the result of joinpoint regression analysis. The number of joinpoints is also shown. Slope values indicate the changes in RD and RR per year (p-value).

**Figure 5.**
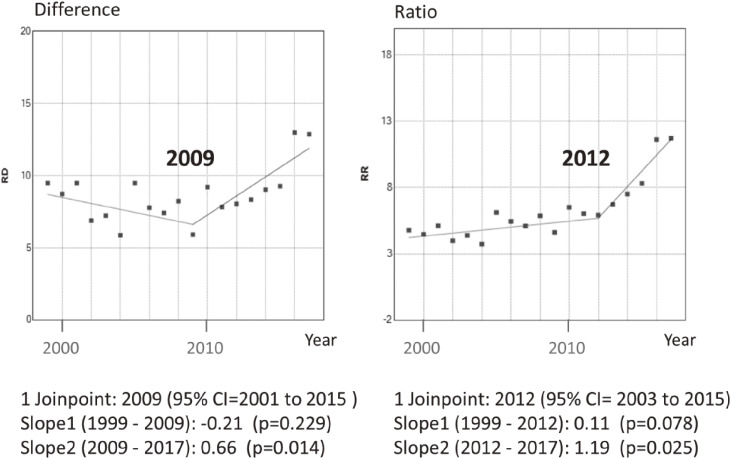
Trends in the Rate Differences (RD) and the Rate Ratios (RR) of infant mortality between unemployed and type II regular worker household in Japan, 1999–2017. The dots indicate each value and the lines indicate the result of joinpoint regression analysis. The number of joinpoints and estimates with 95% confidence intervals (CI) are also shown. Slope values indicate the changes in RD and RR per year (p-value).

**Figure 6.**
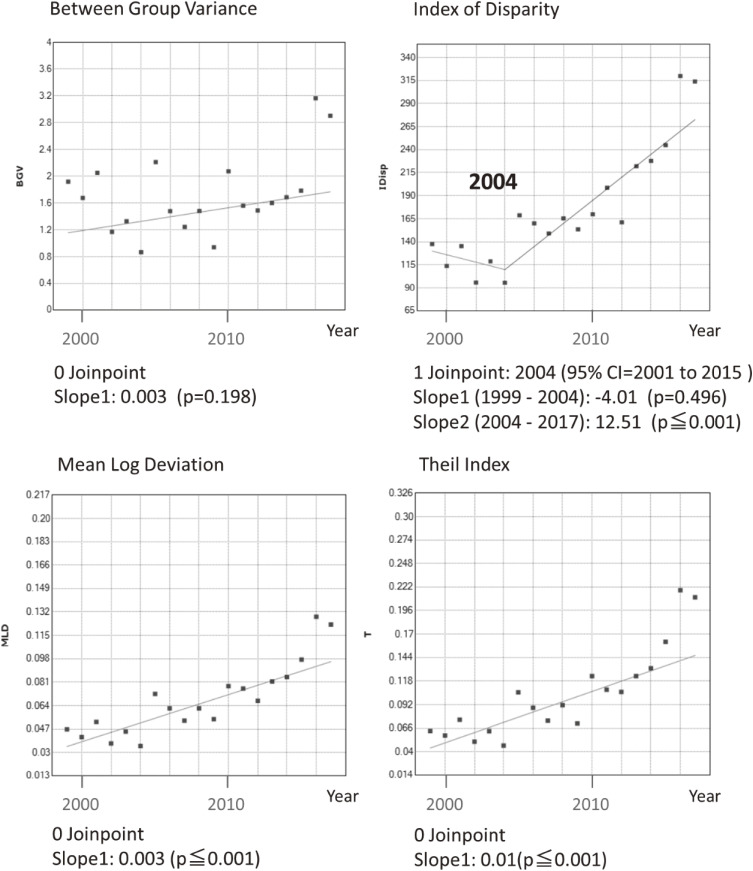
Trends in the various disparity indicators of infant mortality among each occupation of household in Japan, 1999–2017. Between group variance (BGV), Index of Disparity (IDisp), Mean Log Deviation (MLD), and Theil Index (T) are shown. The dots mean each value and the lines mean the result of joinpoint analysis. The number of joinpoint and estimates with 95% confidence intervals (CI) are also shown. Values for slope indicate the changes in each indicator per year (p value).

Joinpoint regression showed a yearly monotonic increase in the differences in the IMR ratios of farming households when compared against type II regular worker households (Figure 4 and eTable 2). Joinpoint regression also indicated that the differences in IMRs between unemployed and type II regular worker households increased sharply from 2009, while a similar divergence in rate ratios was observed from 2012 (Figure 5 and eTable 3). Other absolute measures of inequality, such as between-group variance, and relative measures, such as the index of disparity, mean log deviation, and Theil index, also tended to increase across the observation period. The index of disparity slope was steeper after 2004 (Figure 6 and eTable 4).

## DISCUSSION

Inequality in the IMR increased across household occupation types during the 1999 through 2017 period in Japan. In particular, compared to the IMR of the most privileged occupation type (type II regular workers), overall IMRs nearly doubled (1.96) among farming households and increased 6.5 times among unemployed households. However, there was variability in this phenomenon, as inequality tended to be lower in the prefectures where population density was low and farm density was high.

Our findings on the changing trends in inter-occupational inequality may be closely linked to the health and daily living conditions of women in pregnancy and during child-rearing. Although the data we used did not specify the cause of death, in Japan 30% of infant deaths are due to congenital malformations, 30% occur in the perinatal period, and 10% are due to Sudden Infant Death Syndrome (ie, unexplained infant deaths) or accidents.^24^ In relation to this, it is possible that the high IMRs among farming households may be attributable to the difficulty in resting before and after giving birth. This may stem from the weak job-related legal protections that guarantee maternity and childcare leave in the farming industry.^10^ In Japan, family-run farms constitute 97% of all farms. The possibility of taking maternity and childcare leave for self-employed workers and workers in micro enterprises is reduced when compared to those working for larger companies: there is less financial protection (eg, exemptions from social insurance premiums) for the self-employed and there is the difficulty of finding and paying the wages of proxy workers during leave. The farming industry in Japan has a constant problem with worker shortages, and this difficulty is exaggerated for self-employed households. This shortfall in workers may lead to specific attitudes among pregnant farming women in Japan: they are likely to place their health needs below work and caring for their families.^25^^–^^27^ These challenges for pregnant and childrearing women in farming households may also be due to structural issues, such as gender inequality in farming communities and within households.^28^ Such a link between gender inequality in household decision-making and political participation in the community and infant health has been reported in many other countries as well.^29^^–^^33^

We observed a smaller risk for infant death among farmers in rural and farming prefectures. In those areas, personal connections and the levels of community organizing are likely to be more developed than in urban areas.^34^^–^^36^ Interpersonal relations and organizational partnerships can serve as social capital that facilitates mutual support, diffuses knowledge about healthy behaviors, and spurs collective action leading to more efficacious local policies for those facing daily hardship and whose infants have increased health risks.^6^^,^^37^ These area-level collective factors may help mitigate the adverse effects of the above-mentioned vulnerability in the agricultural working environment and the difficulty women may encounter when trying to participate in decision-making.

The “family management agreement,” which is a governmental program through which public workers help farming households to draw up a written agreement (contract) among family members based on discussions about the household rules for working, might serve as a potential measure to protect the working environment of family farm members.^28^ Although not legally binding, this contract attempts to facilitate farm management while considering the circumstances of each family member.^38^ Since this practice’s inception in around 1995, the number of contracts has gradually expanded each year, from 5,335 households in 1996 to 57,605 in 2018.^28^^,^^39^ However, it may also be beneficial to examine what happens in overseas systems. For example, in France, there is a public social protection system to safeguard farming women during pregnancy and childbirth; labor workers are dispatched to the farm, and the coverage of costs is guaranteed under Agricultural Mutual Social Insurance.^40^^,^^41^

The remarkably high IMRs of unemployed households may be due to socioeconomic hardship and social isolation. In the vital statistics we used, the households with an unemployed status could have been households with one unemployed single parent or households where all the adults were unemployed. In Japan, single parent status is closely linked with poverty: government statistics showed that in 2015, 50.8% of children in single-parent households lived in relative poverty; meanwhile, 10.7% of children in multiple-adult households had a relative poverty status.^42^ This may be important as a recent epidemiologic study in Japan suggested that unemployed status and single parenthood are both risk factors for infant death.^43^ Moreover, the potential effects of poor child health due to poverty and single parenthood may last for years and stretch into later childhood and even adulthood.^44^^,^^45^ As policy changes have gradually reduced social security benefits in Japan since 2013, there is a concern that single mothers may have been more adversely affected by policy reform.^46^

However, the high IMRs of unemployed households may be confounded by health risks; that is, the households with children who have high health risks or mothers with medical problems in pregnancy may be more likely to be unemployed and have a higher risk of infant mortality. This confounding effect may have been magnified in the later years of our observation period, as the unemployment rate declined in Japan after 2010,^47^ and this might also explain the observed trend of increasing occupation-based IMR inequalities after 2010. Households with members who have medical problems may be left behind despite increased opportunities for labor participation. This means that those who are in the unemployed category in the later years of our observation period may be afflicted with greater health risks among their children.

### Limitations

This study has several limitations. First, our unit of analysis, the prefecture, may have been too large to capture regional characteristics accurately. All prefectures in Japan have both urban and rural areas, including the prefectural capital, and areas for farming, timber production, and fishing. Smaller areal units, such as municipality and school districts, may better capture actual local characteristics. Second, when looking at occupations, we used household level statistics; no information was available on each adult member’s actual occupation. Furthermore, the reliability of the measure, “main household occupation,” is questionable. Since there was no formal explanation from the government regarding the definition of the “main household occupation” in the questionnaire, different respondents may have interpreted it in different ways. Some municipalities’ official websites explain that the main household occupation is “the occupation of the head of household,” whereas other municipalities explain it as “the occupation of the person who mainly maintains the household livelihood.” Nonetheless, a family member’s job (eg, farming) could affect all family members due to the job-related culture and community norms. Culture-related explanations, as we have discussed above, can be applied regardless of the variability in occupations among adult family members.

### Conclusion

The existence of inter-occupational inequality in infant mortality within Japanese prefectures—and more importantly, its increase over time—should be studied in greater depth. Doing so will enable the formulation of effective measures to tackle problematic issues in the child-rearing environment; for example, services and infrastructure supporting perinatal parents, the availability of formal and informal support for child-rearing parents, and occupation-specific hazards for safe childbearing. This research should place a special focus on farmers and unemployed households. In addition, this paper highlights the importance of continually evaluating health inequalities according to socioeconomic characteristics. Even if Japan has long had one of the lowest IMRs in the world, the increased inequality in the IMR by household occupation may be affected by local or national politics that have an immediate impact on specific occupations or unemployed people.

## APPENDIX A. SUPPLEMENTARY DATA

eTable 1. The number of infant deaths and births by occupations in each household in Japan, 1999–2017

eTable 2. The rate differences (RD) and the rate ratios (RR) of infant mortality with 95% confidence intervals (CIs) between farming and type II regular worker household in Japan, 1999–2017

eTable 3. The rate differences (RD) and the rate ratios (RR) of infant mortality with 95% confidence intervals (CIs) between unemployed and type II regular worker household in Japan, 1999–2017

eTable 4. The various disparity indicators and standard errors (SEs) of infant mortality with 95% confidence intervals (CIs) among each occupation of household in Japan, 1999–2017
