# Supplementary material for: Infant Mortality Rates for Farming and Unemployed Households in the Japanese Prefectures: An Ecological Time Trend Analysis, 1999–2017
Source: J Epidemiol. 2021 Jan 5;31(1):43–51. doi: 10.2188/jea.JE20190090 (PMC7738643; doi:10.2188/jea.JE20190090)
Supplement: Supplementary file 1 [file je-31-043-s001.pdf]

eTable 1. The number of infant deaths and births by occupations in each household in Japan, 1999–2017

**Year**	**Type II regular worker**		**Type I regular worker**		**Self-employed**		**Farming**		**Other**		**Unemployed**		**Unknown**	
	Death	Birth	Death	Birth	Death	Birth	Death	Birth	Death	Birth	Death	Birth	Death	Birth
**1999**	1,149	479,626	1,186	394,281	349	102,192	162	37,562	623	121,185	281	20,880	251	21,701
**2000**	1,126	484,043	1,140	411,579	344	100,831	129	36,294	530	117,250	360	23,452	193	16,888
**2001**	1,023	465,873	1,063	410,796	305	97,575	136	32,513	508	114,579	298	24,095	261	25,033
**2002**	997	453,415	1,051	408,126	251	94,333	112	29,282	522	111,518	274	25,909	280	31,077
**2003**	925	439,890	1,010	399,611	244	91,039	104	26,622	518	107,168	234	25,181	324	33,929
**2004**	868	437,011	915	396,063	260	88,871	79	25,326	424	102,639	243	24,013	331	36,622
**2005**	780	421,978	911	380,029	241	84,615	77	23,872	417	99,146	286	23,742	239	28,978
**2006**	759	434,752	843	388,695	212	85,822	84	22,212	401	99,705	247	23,232	315	38,080
**2007**	780	437,776	819	387,415	205	84,568	72	20,683	406	95,828	220	21,201	321	42,187
**2008**	751	445,560	786	383,607	193	83,646	58	19,390	383	92,867	246	21,149	376	44,764
**2009**	675	440,130	757	373,133	183	79,674	71	18,639	335	92,130	216	22,761	314	43,469
**2010**	700	448,336	676	373,397	158	78,939	53	18,459	354	96,440	259	24,081	247	31,527
**2011**	650	442,241	729	365,423	170	75,344	65	16,736	340	93,064	240	22,430	264	35,446
**2012**	643	442,915	609	354,512	150	74,066	50	15,536	344	91,608	206	21,023	296	37,504
**2013**	610	447,863	609	347,788	126	74,004	62	14,663	327	88,715	202	19,329	246	37,400
**2014**	559	442,319	578	334,675	147	72,342	43	13,560	303	84,905	193	18,104	255	37,569
**2015**	561	458,419	535	334,744	103	71,771	44	13,147	267	84,501	211	18,535	194	24,507
**2016**	509	448,193	529	319,893	117	69,515	30	11,953	259	80,328	264	16,764	218	30,267
**2017**	492	443,814	485	303,101	104	66,045	29	11,071	250	75,461	196	15,166	202	31,356

eTable 2. The rate differences (RD) and the rate ratios (RR) of infant mortality with 95% confidence intervals (CIs) between farming and type II regular worker household in Japan, 1999–2017

Year	RD	SE	Upper 95% CI	Lower 95% CI	RR	SE	Upper 95% CI	Lower 95% CI
1999	2.35	0.52	3.38	1.33	1.94	0.22	2.42	1.56
2000	1.27	0.46	2.18	0.37	1.51	0.19	1.94	1.17
2001	1.59	0.40	2.38	0.79	1.69	0.19	2.10	1.36
2002	1.21	0.42	2.03	0.39	1.52	0.19	1.95	1.19
2003	1.90	0.44	2.77	1.04	1.89	0.22	2.38	1.50
2004	1.20	0.40	1.98	0.41	1.56	0.19	1.99	1.22
2005	1.31	0.46	2.22	0.40	1.71	0.26	2.30	1.27
2006	2.13	0.55	3.21	1.05	2.22	0.33	2.97	1.66
2007	2.00	0.72	3.41	0.59	2.11	0.41	3.10	1.43
2008	1.76	0.67	3.07	0.45	2.04	0.41	3.01	1.38
2009	2.69	0.70	4.05	1.33	2.65	0.46	3.72	1.89
2010	1.39	0.61	2.59	0.20	1.84	0.38	2.76	1.22
2011	3.12	0.73	4.54	1.69	3.01	0.52	4.21	2.14
2012	1.92	0.73	3.35	0.49	2.18	0.47	3.32	1.43
2013	3.67	0.99	5.61	1.73	3.52	0.73	5.28	2.35
2014	2.57	0.74	4.02	1.13	2.86	0.56	4.21	1.94
2015	2.62	0.82	4.23	1.01	3.06	0.70	4.79	1.96
2016	1.47	0.69	2.82	0.12	2.20	0.58	3.68	1.32
2017	1.02	0.63	2.25	-0.20	1.85	0.54	3.26	1.05

eTable 3. The rate differences (RD) and the rate ratios (RR) of infant mortality with 95% confidence intervals (CIs) between unemployed and type II regular worker household in Japan, 1999–2017

Year	RD	SE	Upper 95% CI	Lower 95% CI	RR	SE	Upper 95% CI	Lower 95% CI
1999	9.48	1.39	12.20	6.76	4.78	0.58	6.07	3.77
2000	8.73	1.05	10.78	6.68	4.47	0.48	5.51	3.62
2001	9.48	0.92	11.28	7.68	5.12	0.46	6.11	4.29
2002	6.89	1.42	9.67	4.11	3.98	0.64	5.47	2.90
2003	7.23	0.98	9.16	5.31	4.39	0.51	5.52	3.50
2004	5.87	0.67	7.18	4.56	3.73	0.35	4.49	3.11
2005	9.49	1.06	11.56	7.41	6.12	0.64	7.50	4.98
2006	7.79	1.04	9.83	5.74	5.46	0.65	6.89	4.32
2007	7.41	1.26	9.88	4.94	5.11	0.76	6.84	3.81
2008	8.23	1.05	10.29	6.17	5.87	0.69	7.40	4.66
2009	5.92	0.83	7.55	4.28	4.63	0.59	5.95	3.60
2010	9.20	1.01	11.18	7.22	6.51	0.78	8.23	5.15
2011	7.82	0.95	9.67	5.96	6.03	0.77	7.74	4.70
2012	8.05	0.82	9.65	6.44	5.93	0.65	7.36	4.78
2013	8.33	1.09	10.48	6.19	6.73	0.90	8.74	5.18
2014	9.03	1.39	11.75	6.31	7.51	1.13	10.08	5.60
2015	9.27	1.23	11.67	6.86	8.30	1.22	11.07	6.23
2016	12.99	1.30	15.55	10.44	11.62	1.29	14.45	9.35
2017	12.87	1.35	15.51	10.22	11.71	1.45	14.94	9.19

eTable 4. The various disparity indicators and standard errors (SEs) of infant mortality with 95% confidence intervals (CIs) among each occupation of household in Japan, 1999–2017

a) Between-group variance (BGV)

Year	BGV	SE	Upper 95% CI	Lower 95% CI
1999	1.92	0.51	2.91	0.93
2000	1.67	0.39	2.43	0.91
2001	2.05	0.39	2.81	1.29
2002	1.17	0.48	2.10	0.24
2003	1.33	0.34	2.00	0.65
2004	0.87	0.19	1.23	0.50
2005	2.21	0.48	3.16	1.27
2006	1.48	0.37	2.21	0.74
2007	1.24	0.40	2.02	0.46
2008	1.48	0.37	2.20	0.76
2009	0.94	0.23	1.40	0.48
2010	2.07	0.45	2.96	1.18
2011	1.56	0.35	2.24	0.88
2012	1.49	0.30	2.07	0.91
2013	1.60	0.39	2.36	0.84
2014	1.69	0.50	2.66	0.71
2015	1.78	0.46	2.68	0.89
2016	3.16	0.63	4.40	1.93
2017	2.90	0.61	4.09	1.72

b) Index of Disparity (IDisp)

Year	IDisp	SE	Upper 95% CI	Lower 95% CI
1999	137.68	17.51	172.01	103.36
2000	113.93	16.32	145.92	81.94
2001	135.33	15.68	166.05	104.60
2002	95.94	18.98	133.15	58.73
2003	118.96	17.19	152.65	85.27
2004	95.81	12.78	120.85	70.77
2005	168.71	20.45	208.78	128.63

2006	160.04	21.33	201.84	118.23
2007	149.17	25.32	198.80	99.54
2008	165.34	23.49	211.37	119.31
2009	153.48	23.75	200.02	106.93
2010	169.81	27.07	222.86	116.76
2011	198.57	30.50	258.34	138.79
2012	161.11	25.34	210.77	111.45
2013	222.05	34.98	290.62	153.49
2014	227.93	36.59	299.65	156.22
2015	244.97	42.69	328.64	161.30
2016	319.89	40.31	398.89	240.89
2017	313.83	45.38	402.77	224.89

c) Mean Log Deviation (MLD)

Year	MLD	SE	Upper 95% CI	Lower 95% CI
1999	0.047	0.008	0.062	0.031
2000	0.041	0.007	0.055	0.027
2001	0.052	0.007	0.066	0.038
2002	0.037	0.010	0.056	0.017
2003	0.045	0.008	0.061	0.029
2004	0.035	0.006	0.046	0.024
2005	0.072	0.010	0.093	0.052
2006	0.062	0.010	0.082	0.042
2007	0.053	0.011	0.075	0.031
2008	0.062	0.010	0.082	0.042
2009	0.054	0.010	0.075	0.034
2010	0.078	0.012	0.103	0.054
2011	0.076	0.013	0.102	0.050
2012	0.067	0.010	0.087	0.048
2013	0.082	0.014	0.109	0.054
2014	0.085	0.016	0.116	0.054
2015	0.097	0.017	0.131	0.064
2016	0.129	0.016	0.160	0.097
2017	0.123	0.018	0.158	0.088

d) Theil Index (T)

Year	T	SE	Upper 95% CI	Lower 95% CI
1999	0.063	0.012	0.087	0.039
2000	0.058	0.011	0.079	0.036
2001	0.075	0.011	0.098	0.053
2002	0.051	0.016	0.083	0.019
2003	0.063	0.013	0.088	0.037
2004	0.047	0.008	0.063	0.030
2005	0.106	0.017	0.139	0.073
2006	0.088	0.017	0.121	0.056
2007	0.074	0.018	0.110	0.039
2008	0.091	0.017	0.125	0.058
2009	0.071	0.015	0.100	0.043
2010	0.123	0.021	0.165	0.081
2011	0.108	0.020	0.147	0.070
2012	0.106	0.017	0.140	0.072
2013	0.123	0.023	0.169	0.078
2014	0.132	0.028	0.187	0.077
2015	0.162	0.031	0.223	0.100
2016	0.218	0.030	0.278	0.159
2017	0.211	0.032	0.273	0.148
